# Improving fatigue resistance of translucent 4Y‐PSZ zirconia through glass gradation

**DOI:** 10.1111/eos.70101

**Published:** 2026-05-03

**Authors:** Felipe Machado Souza, Larissa M. M. Alves, Edisa O. Sousa, Tiago Moreira Bastos Campos, Giovana Assis Marcolino, Mariana Miranda de Toledo Piza, Rainã S. Dias, Satoshi Yamaguchi, Petra C. Gierthmuehlen, Lukasz Witek, Paulo G. Coelho, Estevam A. Bonfante, Ernesto B. Benalcazar‐Jalkh

**Affiliations:** ^1^ Department of Prosthodontics and Periodontology Bauru School of Dentistry University of Sao Paulo Bauru São Paulo Brazil; ^2^ Department of Comprehensive Dentistry University of Maryland School of Dentistry Baltimore Maryland USA; ^3^ Department of Restorative Dentistry School of Dentistry Federal University of Pelotas Pelotas Brazil; ^4^ AI Research Unit Graduate School of Dentistry The University of Osaka Suita Osaka Japan; ^5^ Department of Prosthodontics Medical Faculty and University Hospital Düsseldorf Heinrich‐Heine University Düsseldorf Germany; ^6^ Biomaterials and Regenerative Biology Division NYU College of Dentistry New York New York USA; ^7^ Department of Biomedical Engineering New York University Tandon School of Engineering Brooklyn New York USA; ^8^ Department of Oral and Maxillofacial Surgery NYU College of Dentistry New York New York USA; ^9^ Hansjörg Wyss Department of Plastic Surgery New York University Grossman School of Medicine New York New York USA; ^10^ DeWitt Daughtry Family Department of Surgery Division of Plastic Surgery University of Miami Miller School of Medicine Miami Florida USA; ^11^ Department of Biochemistry and Molecular Biology University of Miami Miller School of Medicine Miami Florida USA

**Keywords:** fatigue, glass infiltration, reliability, SSALT, translucent zirconia

## Abstract

To evaluate the effect of graded glass infiltration on the fatigue behavior and mechanical reliability of translucent 4Y‐PSZ zirconia before and after hydrothermal aging, disc‐shaped specimens were fabricated by uniaxial pressing and divided into control and glass‐graded groups (*n* = 36/group). Glass infiltration was performed on pre‐sintered specimens followed by final sintering, and half of the specimens from each group underwent hydrothermal aging (134°C, 2.2 bar, 20 h). Microstructure and phase composition were assessed by scanning electron microscopy and x‐ray diffraction. Mechanical performance was evaluated using step‐stress accelerated life testing, with Weibull statistics, reliability analysis, and inverse power‐law modeling. Glass‐graded specimens demonstrated higher reliability and characteristic strength (≈330 MPa increase) with similar Weibull modulus compared to controls. The inverse power‐law parameter *α*_0_ was higher for the glass‐graded group, indicating extended fatigue life, whereas comparable *α*_1_ values suggested similar life–stress relationships. Hydrothermal aging did not significantly affect mechanical performance, although phase transformation occurred in the control group. Fractography revealed surface‐initiated failures in controls and interface‐related crack initiation in glass‐graded specimens. Graded glass infiltration improved the fatigue reliability and characteristic strength without compromising hydrothermal stability of 4Y‐PSZ. These results suggest that glass‐graded 4Y‐PSZ may expand the clinical applicability of translucent zirconia for long‐span (≥4‐unit) prosthetic reconstructions.

## INTRODUCTION

Among the various generations of dental zirconia (ZrO_2_) currently available, translucent zirconias have gained increasing attention due to their improved optical properties and enhanced resistance to low‐temperature degradation (LTD). These characteristics are primarily associated with the incorporation of higher concentrations of stabilizers, such as yttrium oxide (>4 mol% Y_2_O_3_), which promote the formation of a significant fraction of the cubic crystalline phase [1, 2]. The isotropic nature of cubic grains reduces light scattering, thereby increasing translucency, and contributes to improved resistance to hydrothermal aging, as the cubic phase does not undergo the tetragonal‐to‐monoclinic transformation typically observed in conventional 3Y‐TZP [3, 4, 5, 6].

Historically, first‐generation zirconia (3Y‐TZP) was characterized by high opacity and therefore indicated mainly as a framework material, requiring veneering with feldspathic ceramics to meet esthetic demands. However, bilayered restorations have been associated with clinical complications, particularly chipping and delamination of the veneering layer [7, 8, 9]. In response, subsequent generations of zirconia were developed to improve translucency and enable their indication as monolithic restorations. Although processing modifications and reductions in alumina content resulted in modest optical improvements, these changes also increased susceptibility to LTD and did not fully overcome esthetic limitations [10, 11]. Moreover, clinical and laboratory investigations have demonstrated that 3Y‐TZP may exhibit structural degradation over time when exposed to humid environments [12, 13, 14].

Increasing yttria content led to the development of partially stabilized zirconias, such as 5Y‐PSZ, which may contain more than 50% cubic phase and exhibit superior translucency. However, the predominance of the cubic phase suppresses the transformation toughening mechanism, resulting in a substantial reduction in mechanical strength, typically ranging from 600 to 800 MPa [3, 15]. In this context, 4Y‐PSZ zirconia has emerged as an intermediate alternative, combining a significant cubic‐phase fraction with a higher proportion of tetragonal grains. This composition provides a more favorable balance between optical and mechanical properties, with flexural strength values generally reported between 600 and 1000 MPa [11, 16, 17]. Nevertheless, when compared to conventional 3Y‐TZP, which may exceed 1000 MPa in flexural strength, 4Y‐PSZ still exhibits inferior mechanical performance, which may limit its indication in load‐bearing applications, including long‐span fixed dental prostheses, FDP (≥4 units, ISO 6872 Class 5 ceramics).

In parallel, multilayered zirconia systems have been introduced as an alternative strategy to reconcile esthetics and mechanical performance by combining layers with different yttria contents. Although these systems provide improved optical gradients and mechanical properties, their performance remains dependent on factors, such as layer configuration, thickness distribution, and correct positioning during milling, which may introduce variability in clinical outcomes [1, 18, 19]. Furthermore, monolithic restorations remain desirable, as they reduce the risk of veneer‐related complications previously reported in bilayered systems [20, 21, 22, 23]. Consequently, the development of monolithic zirconia materials that combine high translucency with improved mechanical reliability continues to be of great interest in dental materials research.

One promising strategy to enhance the mechanical performance of zirconia without compromising optical properties is glass infiltration. In this approach, glass is infiltrated into a porous or partially sintered zirconia substrate, producing a graded glass–ceramic structure that may modify surface flaw populations, improve crack resistance, and enhance fatigue behavior [22, 24, 25]. Previous investigations have demonstrated that glass infiltration in 3Y‐TZP may reduce surface roughness and increase flexural strength, suggesting that graded glass–zirconia structures can effectively improve mechanical performance [24]. Preliminary evidence also indicates that similar approaches may benefit more translucent zirconias, although the available data remain limited.

Considering that 4Y‐PSZ zirconia provides a favorable balance between translucency and strength, it may represent a particularly suitable candidate for glass infiltration, potentially enabling mechanical performance closer to that of high‐strength zirconias, thus allowing its safer indication as ≥4‐unit U FDP while preserving optical properties required for monolithic restorations. However, the influence of glass infiltration on the fatigue behavior of 4Y‐PSZ zirconia remains largely unexplored in the current literature. Therefore, the aim of the present study was to develop a glass‐infiltrated 4Y‐PSZ zirconia and to evaluate its mechanical performance under cyclic fatigue conditions, comparing it with non‐infiltrated controls before and after hydrothermal aging. The null hypothesis tested was that glass infiltration would not result in a statistically significant improvement in the fatigue behavior of 4Y‐PSZ zirconia.

## MATERIAL AND METHODS

### Sample preparation and glass gradation

A 4Y‐PSZ commercially available high‐purity powder was used in the present study (Zpex4, Tosoh). A total of 72 specimens were synthesized and divided into two experimental groups: control nongraded and glass‐graded (*n* = 36 per group). For sample preparation, 0.9 g of 4Y‐PSZ powder was weighed and placed into a tempered steel carbide matrix. Disc‐shaped samples were fabricated by uniaxial pressing using a hydraulic press (MAO098, Marconi) at 3 t of pressure for 30 s. All specimens were pre‐sintered at 1000°C for 1 h in a high‐temperature furnace (Nabertherm).

The non‐infiltrated group samples (*n* = 36) were sintered at 1550°C with a heating and cooling rate of 4°/min, as recommended by the manufacturer. After sintering, one surface of each sample was polished using a semiautomatic polisher (Automet 2000, Buehler) with a complete sequence of diamond discs and suspensions up to 1 *µ*m (ALLIED High‐Tech Products). The opposite surface was ground flat to produce disc‐shaped samples with a diameter of 12 mm and a thickness of 1.2 ± 0.2 mm, in accordance with ISO 6872:2015. The remaining samples (*n* = 36) were infiltrated with glass following the methodology described below.

The glass used for gradation was obtained by the extraction of silicic acid by passing sodium metasilicate (Na_2_SiO_3_·5H_2_O) in aqueous solution (10% w/w) through an ion exchange resin (IR120—Rohm and Haas). Aluminum (Al_2_O_3_—11.7%), calcium (CaO—3.0%), sodium (Na_2_O—7.3%), potassium (K_2_O—10.0%), and boron (B—4.0%) salts were then added to form the glass. This composition was obtained by mixing boric acid corresponding to the desired cations and the silicic acid solution for the silica component. The material was then kept in an oven at 100°C for 24 h and subsequently calcined at 600°C for 5 h. The resulting glass was ground and sieved (200 mesh). The glass was mixed with propylene glycol and applied to the pre‐sintered samples using a flat‐bristle synthetic brush. After application and drying, the final sintering was performed at 1550°C for 2 h with a heating and cooling rate of 4°/min.

Subsequently, the microstructure and crystalline composition of representative samples of both groups were characterized by scanning‐electron microscopy (SEM) and x‐ray diffraction (XRD), respectively, and the fatigue reliability of both groups was assessed by step‐stress accelerated life testing before and after hydrothermal aging followed by fractographic evaluations.

### Aging

Half of the samples of each group were subjected to accelerated hydrothermal aging, which was performed in a hydrothermal reactor at 134°C under 2.2 bar pressure for 20 h with samples submerged in water. The aging parameters were based on a systematic literature review [10], which suggests that this time and temperature are sufficient to induce phase transformation. Additionally, the protocol followed previously published studies that demonstrated that the use of a hydrothermal reactor provides consistent pressure and temperature conditions throughout the aging process [26, 27, 28, 29], particularly for 4Y‐PSZ ceramics [30].

### Scanning electron microscope (SEM)

The surface micromorphology of the ceramic systems was analyzed through field‐emission gun SEM (Tescan Vega3). All SEM evaluations were conducted on specimens in the as‐processed condition (post‐sintering and after hydrothermal aging), without any additional thermal or chemical treatment prior to analysis, to preserve the original surface characteristics. Cross‐sectioned samples of each group were also evaluated before and after aging to depict degradation depth in aged groups [29, 30]. SEM micrographs were obtained using secondary and backscattered electron detectors, under high vacuum conditions, with an accelerating voltage of 5 kV and magnifications up to 20,000×.

### X‐ray diffraction (XRD)

The crystalline structure of both groups was analyzed by XRD (X'Pert Power PANalytical, the Netherlands) before and after hydrothermal aging. The scanning was performed on the Bragg *θ*–2*θ* geometry, equipped with a graphite monochromator and Cu *K*α radiation (*λ* = 1.5406 Å), operating at a voltage of 40 kV and a current emission of 40 mA. The data were obtained in periods of 1.0 s and steps of 0.020 (2*θ*) of 20°–70°. Baseline subtraction was performed in highscore plus Software (Malvern Panalytical) for all XRD‐collected data. Monoclinic and tetragonal peaks were identified, and the area corresponding to peaks 28°, 30°, and 31.2° was recorded for monoclinic phase calculation. The monoclinic phase percentage was calculated after aging through the formulas introduced by Toraya and Yoshimura [31].

### Step stress accelerated life testing (SSALT)

For step stress accelerated life testing (SSALT), disc‐shaped specimens were subjected to cyclic loads, with successively increasing stress levels, until their failure or suspension [32]. For testing, specimens were positioned on a piston‐on‐three‐balls biaxial flexural strength test device in an all‐electric precision fatigue system according to ISO 6872:2015 requirements (ElectroPuls E3000 Linear Torsion system, Instron) and tested under fatigue at 15 Hz in humid, room‐temperature environment (∼23°C). SSALT was performed until failure or suspension of the specimens using three different load profiles, where samples were distributed in a ratio of 3:2:1 to mild (*n* = 9), moderate (*n* = 6), and aggressive (*n* = 3) profiles, respectively, as described in previous literature [32, 33, 34]. The stress (MPa) at fatigue failure was calculated using the formula provided in ASTM F 394‐78:

(1) $$S=-0.238P\;\frac{\left(X-Y\right)}{d^{2}}$$

where *S* is the biaxial flexural strength, *P* is the load at failure, *d* is the specimen thickness (1.2 mm), and *X* and *Y* were determined according to the following equations:

(2) $$X=\left(1+\nu \right)\ln{\left(\frac{B}{C}\right)}^{2}+\left[\frac{\left(1-\nu \right)}{2}\right]{\left(\frac{B}{C}\right)}^{2}$$

(3) $$Y=\left(1+\nu \right)\left[1+\ln{\left(\frac{A}{C}\right)}^{2}\right]+\left(1-\nu \right){\left(\frac{A}{C}\right)}^{2\;}$$

where *v* is Poisson's ratio (0.31), *A* is the radius of the circle formed by the supporting balls (5.5 mm), *B* is the radius of the piston tip (0.75 mm), and *C* is the radius of the specimen (6 mm). The outcomes were recorded as fracture resistance, stress profile, and number of cycles at which each specimen failed.

**FIGURE 1:**
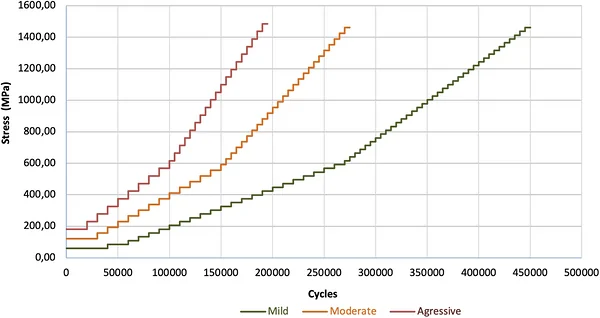
Fatigue profiles: mild, moderate, and aggressive, utilized for step‐stress life testing data.

For SSALT data evaluation, load values of fatigue profiles were converted to stress employing Equations ((1), (2), (3)) and inputted to alta pro v.21 software (Reliasoft), as presented in Figure 1.

Based upon the step‐stress distribution of failures, the inverse power law life‐stress relationship for damage accumulation and Weibull distribution [35] model enabled the calculation and plot of the use‐level probability Weibull curves, represented by probability of failure versus number of cycles with the corresponding 95% two‐sided confidence interval (CI) (alta pro v.21; Reliasoft). Fatigue damage accumulation was modeled using the inverse power law relationship, in which the stress‐life behavior is described as a function of the parameters *α*_0_ and *α*_1_. This model assumes that the number of cycles to failure decreases as a power function of applied stress and has been widely used in step‐stress accelerated life testing of brittle materials. Parameter estimation and corresponding 95% confidence bounds were calculated using maximum likelihood methods (alta pro v.21), adopting two‐sided CIs of 95%. The stress at failure obtained from SSALT represents the residual strength of specimens after cumulative fatigue damage and was also treated as a strength distribution for Weibull analysis.

The use of level probability in Weibull analysis also provides a beta (*β*) value that describes the failure rate behavior over time. When failure rate decreases over time (*β* < 1), it is commonly associated with early failures. When failure rates increase over time (*β* > 1), it is related to damage accumulation and fatigue, and when beta is equal to one (*β* = 1), it represents failures of a random cause. The reliability, which represents the probability of an item surviving a given number of cycles at a use‐level stress, was calculated for a mission of 100,000 cycles at 100, 300, 500, and 800 MPa (stress requirements described in ISO 6872:2024 for ceramic materials). For the mission reliability and *β* parameters calculated, the two‐side 95% CIs were calculated. As *β* was lower than 1 for a determined group, Weibull probability contour plot, represented by the Weibull modulus versus the characteristic strength, was calculated and plotted, where contour limits were determined by the calculation of the two‐sided 95% CI.

### Fractographic analyses

The fractured specimens were examined with polarized light in Axio Zoom V16 Stereo Zoom Microscope (Carl Zeiss) to assess fractographic evidence of the fracture origin and direction of propagation. The Z‐stack imaging mode was used to merge images automatically taken on the z‐plane into one figure with improved depth of focus (zen 2.3 pro, Zeiss).

## RESULTS

**FIGURE 2:**
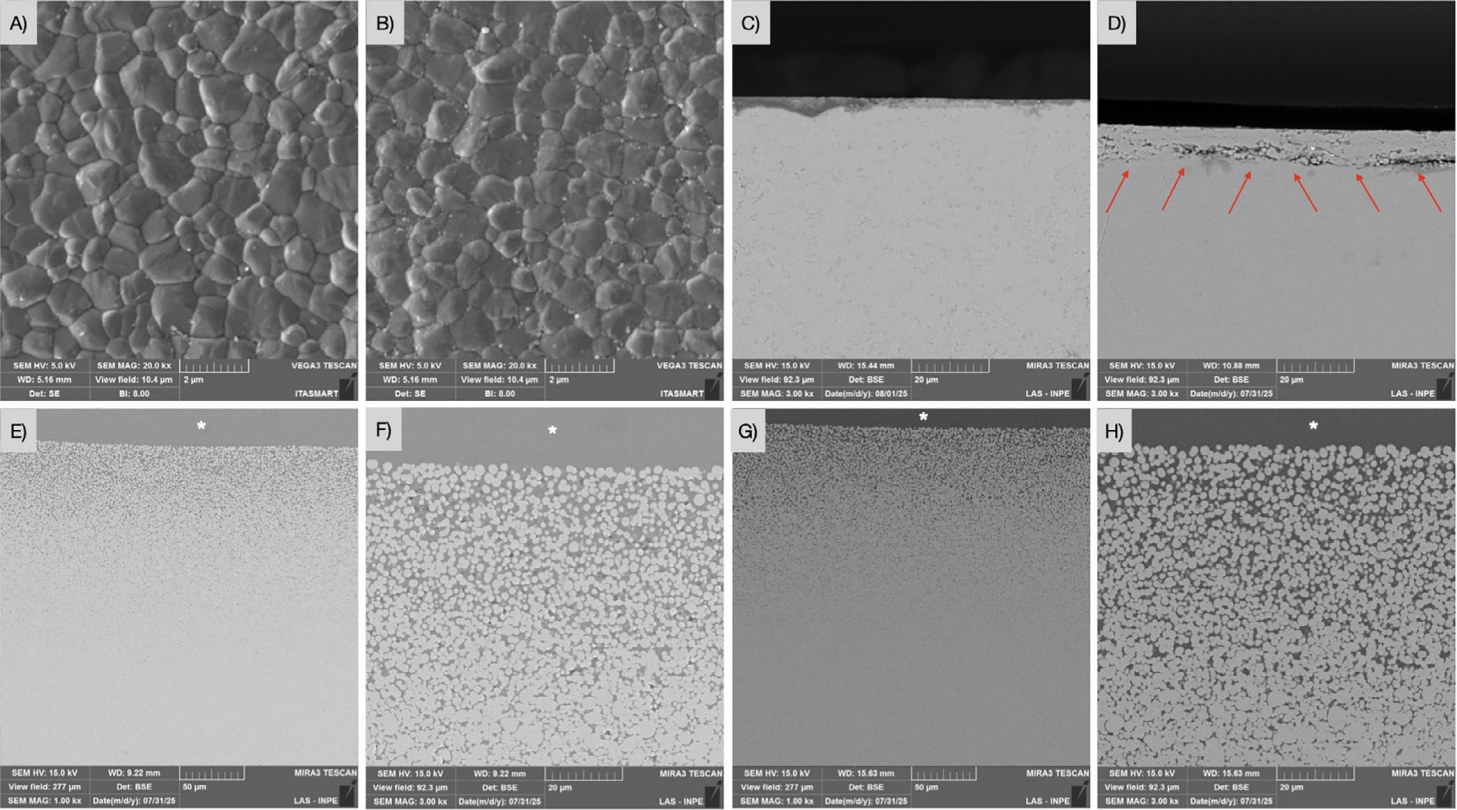
Surface scanning electron microscope (SEM) micrographs of the synthesized 4Y‐PSZ before (A) and after aging (B). Parts (C) and (D) present the cross‐section micrographs of the control group before and after aging, respectively, where red arrows point to the bottom of the degraded layer. Parts (E) and (F) are cross‐section micrographs of the glass‐graded group before aging in low and high magnifications, respectively. Parts (G) and (H) present the cross‐section micrographs of the glass‐graded group after aging in low and high magnifications, respectively. White asterisks mark the glass surface of the infiltrated groups.

SEM surface evaluation of nongraded groups is presented in Figure 2. Surface micrographs of control 4Y‐PSZ (Figure 2A) revealed a dense microstructure with regular grains and minimal microstructural defects originated from the ceramic processing. Moreover, no significant microstructural changes were observed in 4Y‐PSZ after the accelerated hydrothermal aging protocol (Figure 2B). Cross‐section SEM images of the control group demonstrated the presence of a degradation layer in the surface after aging (Figure 2D) with a depth of approximately 10 µm. The cross‐section images of the glass‐graded group depict the formation of a graded area of approximately 50 µm, with no significant changes after hydrothermal aging.

**FIGURE 3:**
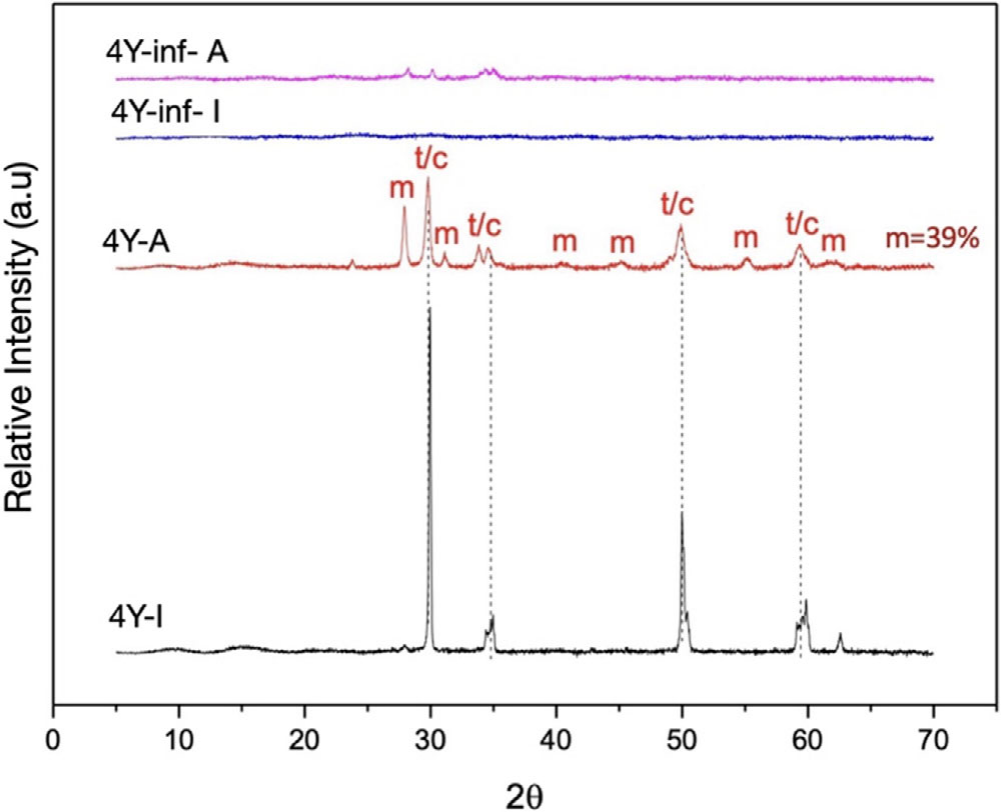
Diffractograms of the non‐infiltrated experimental group before (4Y‐I) and after hydrothermal aging (4Y‐A). Peak evaluation using the method by Toraya *et al.* indicated the presence of 39% monoclinic phase content after aging. In contrast, the diffractograms of the glass‐infiltrated groups did not show crystalline peaks due to the presence of glass on the surface of the samples.

XRD evaluations depicted characteristic tetragonal and cubic peaks in the 4Y‐PSZ immediate condition (Figure 3). After aging, significant phase transformation, reaching 39% of monoclinic content, was observed in the control group. For the glass‐infiltrated groups, no discernible zirconia crystalline peaks were detected at the surface due to attenuation by the glass layer, as shown in Figure 3.

**FIGURE 4:**
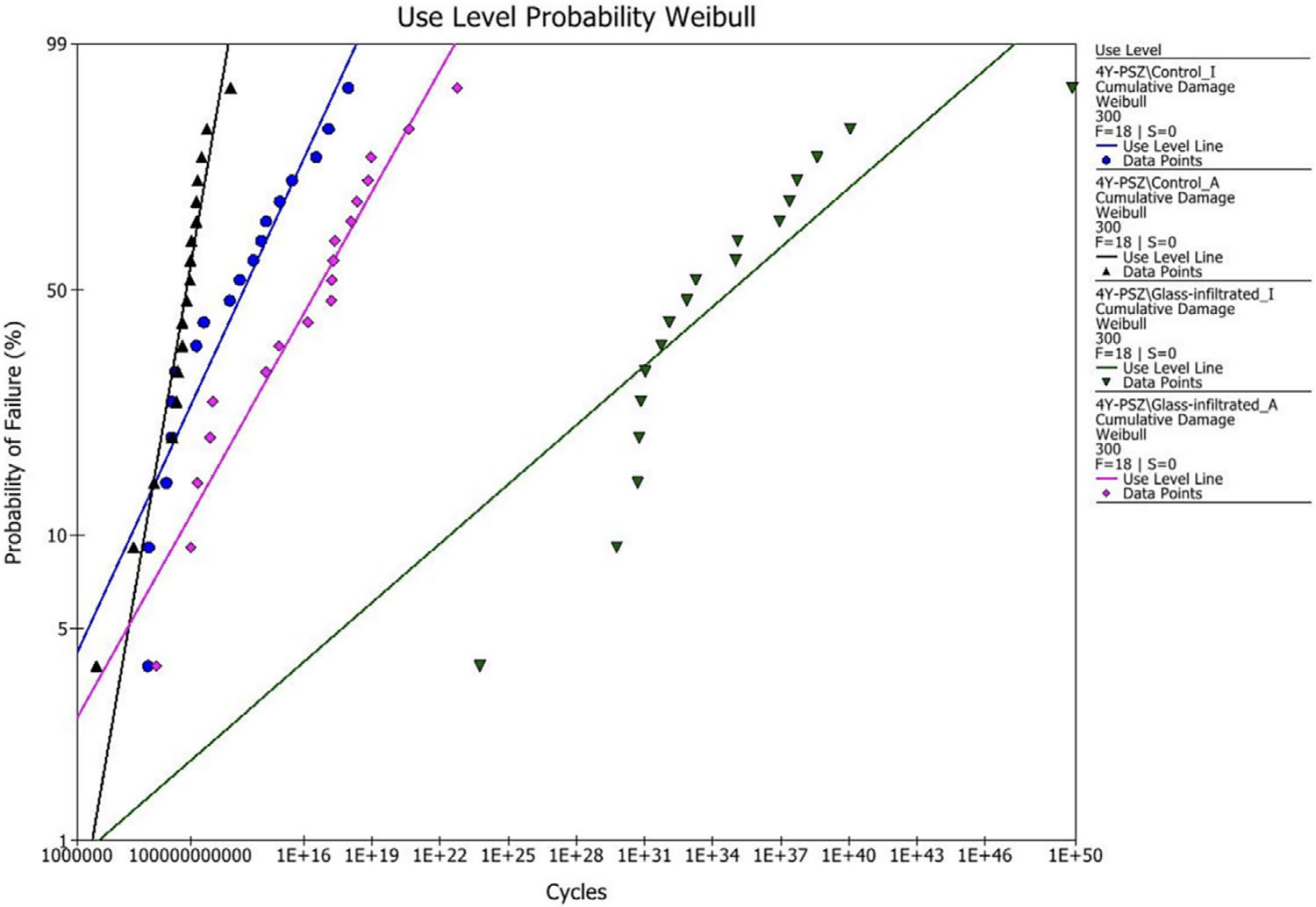
Weibull probability plot showing the probability of failure for the tested samples as a function of the number of cycles at a set stress of 300 MPa.

The inverse power law model parameters *α*_0_ and *α*_1_ were also obtained from the fatigue analysis. The control group presented *α*_0_ values of 48.3 (95% CI: 11.8–84.7) in the immediate condition and 38.3 (95% CI: 9.4–67.3) after hydrothermal aging, whereas the glass‐infiltrated group showed *α*_0_ values of 112.2 (95% CI: 55.0–169.5) in the immediate condition and 54.4 (95% CI: 9.8–99.1) after aging. The *α*_1_ parameter presented negative values for all experimental groups, with values of −0.051 (95% CI: −0.099 to −0.004) and −0.042 (95% CI: −0.084 to −0.0002) for the control group (immediate and aged, respectively), and −0.088 (95% CI: −0.137 to −0.040) and −0.045 (95% CI: −0.090 to −0.0009) for the glass‐infiltrated group (immediate and aged, respectively). The analysis of the fatigue test results using Weibull distribution (Figure 4) also yielded *β* values < 1 (0.16 and 0.06 for the immediate control and glass‐infiltrated groups, respectively; and 0.44 and 0.13 for the aged control and infiltrated groups), suggesting that failures were dictated by the material's strength [32, 33]. The obtained *β* values enabled the application of a two‐parameter Weibull analysis to determine the characteristic strength and Weibull modulus of the experimental groups, as presented in Table 1.

**TABLE 1:** Weibull parameters for the control and glass‐infiltrated groups with corresponding 95% confidence intervals under immediate and aged conditions considering fatigue fracture load values transformed in stress.

	Control		Glass‐graded	
	Immediate	Aged	Immediate	Aged
Upper bound	843.9	741.7	1180.3	1100.1
**Characteristic strength (MPa)**	782.5 Bb	714.6 Bb	1134.2 Aa	1014.3 Aa
Lower bound	725.5	688.6	1089.94	935.3
Upper bound	9.3	18.5	17.4	9.3
**Weibull modulus**	6.5 Aa	13 Aa	12.3 Aa	5.9 Aa
Lower bound	4.5	9.1	8.7	4.1

*Note*: Different uppercase letters denote statistically significant differences between groups under the same condition. Different lowercase letters denote statistically significant differences before and after aging for the same experimental material.

**FIGURE 5:**
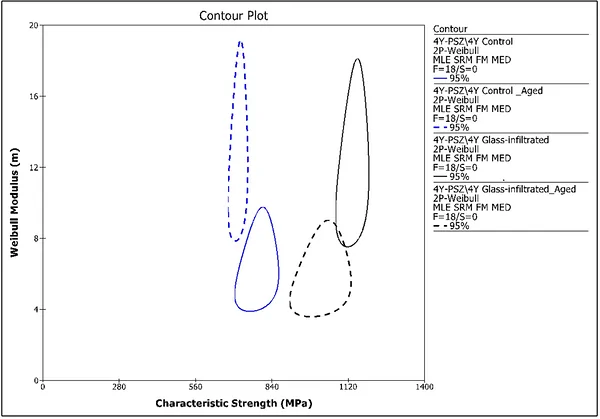
Contour plot as a function of characteristic strength and Weibull modulus. The absence of contour overlap indicates statistical differences between the aged groups.

The glass gradation protocol significantly increased the characteristic strength of the ultra‐translucent 4Y‐PSZ zirconia in ∼330 MPa. This relationship is clearly illustrated in the contour plot (Figure 5), where the absence of contour overlap demonstrates statistically significant differences in strength between the control and infiltrated groups. Despite the increase in characteristic strength, no statistically significant differences were observed in the Weibull modulus between the groups. After aging, the statistical difference between the experimental groups remained; however, aging did not lead to significant changes in strength for the experimental materials.

Survival probability evaluations for missions of 100,000 cycles under clinically relevant loads, as reported in ISO 6872, were performed and are presented in Table 2. Both the control group and the glass‐graded material presented nearly 100% reliability at 100 MPa. For missions at 300 and 500 MPa, the immediate survival probability of the glass‐graded group was significantly higher compared to the control group. Finally, at 800 MPa, both experimental groups experienced a significant drop in survival probability; however, glass‐graded groups presented higher reliability compared with nongraded control groups.

**TABLE 2:** Survival probability of the experimental groups with 95% confidence intervals for missions of 100,000 cycles at loads of 300, 500, and 800 MPa.

	Control 4Y‐PSZ		Glass‐graded 4Y‐PSZ	
100,000 cycles	Immediate	Aged	Immediate	Aged
Upper bound	97	99	99	99
**Reliability at 300** **MPa**	**90 B**	**99 A**	**99 A**	**98 A**
Lower bound	71	99	99	92
Upper bound	77	97	99	97
**Reliability at 500** **MPa**	**54 B**	**92 A**	**99 A**	**93 A**
Lower bound	24	78	96	83
Upper bound	40	7	89	80
**Reliability at 800** **MPa**	**0 B**	**3 B**	**76 A**	**67 A**
Lower bound	0	0	53	47

*Note*: Different letters denote significant differences between the groups.

**FIGURE 6:**
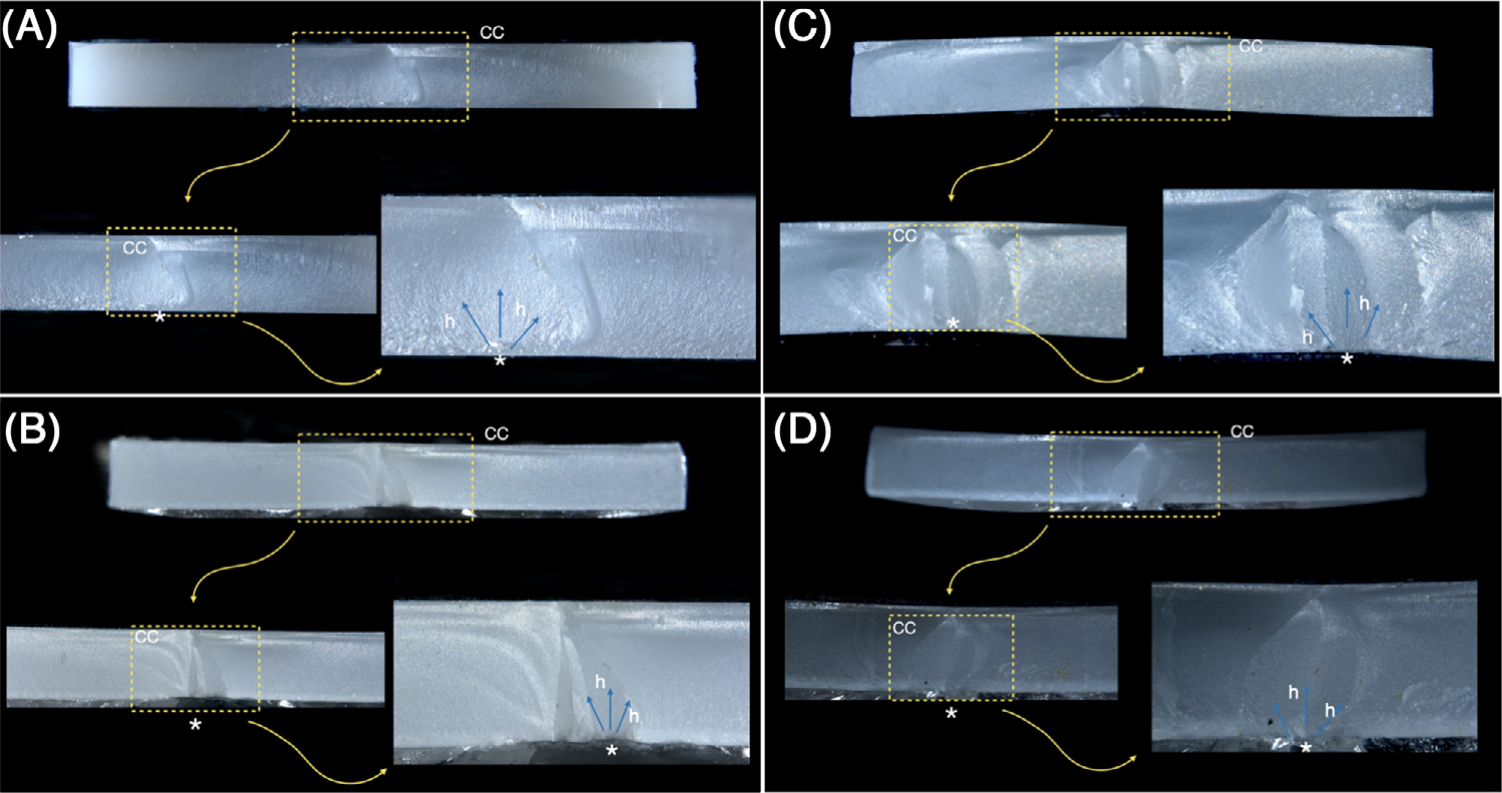
Fractographic evaluation of representative samples from the experimental groups: (A) non‐infiltrated 4Y‐PSZ, (B) glass‐graded 4Y‐PSZ, (C) aged non‐infiltrated 4Y‐PSZ, and (D) aged glass‐infiltrated 4Y‐PSZ. The compression curl (CC) was used to identify tensile and compressive surfaces, and hackle lines (h) allowed the suggestion of the fracture origin (*) and direction of crack propagation (blue arrow).

Fractographic evaluation using a stereomicroscope allowed us to suggest the origin and propagation of fractures in the experimental groups. Figure 6 depicts representative images from both the control and the glass‐infiltrated groups. In the non‐infiltrated groups, the fracture origin could be traced to surface defects under tensile stress during the fatigue test, which propagated toward the compressive surface due to mechanical loading. In contrast, for the glass‐infiltrated groups, the fracture likely originated from defects at the interface between the zirconia surface and the glass layer, from which the crack propagated toward the compressive surface.

## DISCUSSION

The present study evaluated the effect of graded glass infiltration on the fatigue behavior and reliability of 4Y‐PSZ zirconia before and after hydrothermal aging. The results demonstrated that glass gradation significantly increased the characteristic strength and survival probability of the material under cyclic loading, while maintaining similar Weibull modulus values compared with the non‐infiltrated control group. In addition, XRD and SEM analyses indicated that the glass‐graded specimens exhibited reduced susceptibility to hydrothermal aging, in contrast to the control group where a transformed layer and increased monoclinic content were observed after aging. Fractographic evaluation further revealed differences in fracture initiation patterns between groups, suggesting that glass infiltration modified the dominant failure mechanisms under fatigue loading. Therefore, the null hypothesis that graded glass infiltration would not improve the fatigue behavior of 4Y‐PSZ zirconia was rejected.

Combining improved esthetics and greater resistance to LTD compared to 3Y‐TZP [1], along with superior mechanical properties relative to 5Y‐PSZ [11], 4Y‐PSZ zirconia stands out as a promising material for broader use in monolithic applications. The synthesis protocol used in this study resulted in dense ceramic surfaces with regular grain distribution and no evidence of residual porosity, confirming adequate consolidation and sintering of the 4Y‐PSZ material. For the glass‐graded specimens, cross‐sectional SEM imaging revealed the formation of a graded region comparable to that previously reported for glass‐infiltrated 3Y‐TZP [36, 37] and 5Y‐PSZ systems [25, 38], indicating effective penetration of the glass phase into the superficial microstructure. The formation of this graded layer is known to modify the near‐surface stress field and flaw population by filling intergranular spaces and reducing the size and severity of critical surface defects, which are the primary sites for crack initiation in brittle ceramics [25, 39, 40]. In addition, the presence of a glass‐rich surface region may promote a gradual transition in elastic modulus, contributing to stress redistribution and a reduction in tensile stress concentration at the surface, which is consistent with previous studies on graded zirconia structures [25, 39, 41, 42]. Within the limitations of the qualitative SEM analysis, the glass‐infiltrated layer appeared homogeneous across the evaluated cross‐sectional regions and among different specimens, with no evidence of localized infiltration defects or interfacial discontinuities. This observation supports the reproducibility of the infiltration process and is consistent with previous reports on glass‐modified zirconia systems [25, 39, 41, 42, 43]. However, the absence of a quantitative assessment of the compositional and mechanical gradients represents a limitation of the present study.

XRD analysis demonstrated the characteristic crystalline structure of 4Y‐PSZ, with the presence of both tetragonal and cubic phases in the immediate condition. After hydrothermal aging, the control group exhibited measurable monoclinic phase content, confirming that 4Y‐PSZ remains susceptible to LTD due to the presence of metastable tetragonal grains, as has been previously reported [30]. In contrast, no crystalline peaks could be clearly detected in the glass‐infiltrated group after aging, likely due to attenuation of diffraction by the glass layer, which prevented direct surface evaluation of phase transformation. It is noteworthy that XRD evaluations present inherent limitations when applied to partially stabilized zirconias, including its limited penetration depth, typically restricted to a few micrometers, such that the quantified monoclinic content primarily reflects the transformed surface layer rather than the bulk material [31]. Additionally, the significant overlap between tetragonal and cubic diffraction peaks complicates phase discrimination, particularly in mixed‐phase systems such as 4Y‐PSZ and may affect the accuracy of intensity‐based quantification. These limitations are further accentuated in the glass‐infiltrated group, where the presence of the glass layer attenuates the diffraction signal and hinders direct assessment of the underlying zirconia phases. Nevertheless, cross‐sectional SEM provided indirect evidence of the protective effect of glass infiltration. Although the control specimens exhibited a degraded surface layer of approximately 10 µm after aging, no measurable microstructural alterations were observed in the graded specimens before or after aging. These findings suggest that the infiltrated glass layer may act as a barrier to water diffusion, limiting the progression of hydrothermal degradation and preserving the microstructural integrity of the zirconia substrate [44].

The improvement in mechanical performance observed in the present study was primarily reflected by the significant increase in characteristic strength (∼330 MPa) and survival probability of the glass‐graded groups, whereas Weibull modulus values remained within a similar range. This behavior suggests that glass infiltration did not substantially alter the variability of the material but rather shifted the strength distribution toward higher stress levels, indicating a reduction in the severity of critical flaws. In brittle ceramics, fatigue failure is predominantly governed by subcritical crack growth originating from surface defects, and small changes in flaw size or morphology may lead to significant increases in fatigue resistance [45, 46]. The graded glass layer may contribute to this effect by reducing stress intensity at crack tips and promoting crack deflection or bridging at the glass–zirconia interface, mechanisms that have been previously associated with enhanced fatigue resistance in graded ceramic systems [25, 39]. Furthermore, the elastic modulus gradient generated by the infiltrated layer may reduce tensile stress concentration at the surface, which is the region subjected to maximum tensile stresses during biaxial fatigue testing [42]. This combination of reduced flaw severity and modified stress distribution provides a plausible explanation for the substantial increase in characteristic strength and reliability observed for the glass‐graded specimens under cyclic loading.

In addition to the Weibull parameters and characteristic strength, the inverse power law parameters *α*_0_ and *α*_1_ provided complementary information regarding fatigue behavior. The parameter *α*_0_, which is associated with the characteristic fatigue life under the tested conditions, was higher for the glass‐infiltrated specimens in the immediate condition, indicating a longer expected lifetime under cyclic loading [34, 35]. This observation is consistent with the increase in characteristic strength and survival probability, suggesting that glass infiltration delayed subcritical crack growth by reducing the severity of surface flaws. The parameter *α*_1_, which describes the sensitivity of fatigue life to load amplitude, presented similar values among the experimental groups, indicating that the fundamental life–stress relationship was not substantially altered by glass infiltration or hydrothermal aging. These results suggest that the graded structure primarily shifted the fatigue life distribution toward higher characteristic lives without significantly changing the fatigue damage kinetics.

The survival probability results observed in the present study are particularly relevant from a clinical perspective, as they indicate that the mechanical limitations traditionally associated with translucent zirconias [1, 11, 47] may be partially overcome through glass gradation. Both materials presented high reliability at lower stress levels, compatible with the indication of single crowns. However, at stress levels of 300 and 500 MPa, corresponding to conditions commonly associated with fixed dental prostheses in anterior and posterior regions, respectively [48], the glass‐graded material exhibited significantly higher survival probability compared with the control group. At 800 MPa, a stress level that corresponds to the minimum strength requirement established by ISO 6872:2024 for ceramic materials indicated for long‐span fixed dental prostheses, the graded material maintained substantially higher reliability, whereas the control group showed a pronounced reduction in survival probability. These findings suggest that modifying the surface and near‐surface structure of 4Y‐PSZ through glass infiltration may expand its clinical applications to long‐span and full‐arch dental prostheses (≥4 U, Class 5 ceramics, ISO 6872). Although further studies using anatomical geometries and more complex loading conditions are necessary, the present results suggest that graded 4Y‐PSZ may represent a promising approach to increase the mechanical reliability of ultra‐translucent zirconias for demanding monolithic clinical applications.

Interestingly, hydrothermal aging did not significantly affect the mechanical performance of either group, despite the clear evidence of phase transformation and surface degradation. This finding was expected for the glass‐graded material, in which the graded structure was designed to mitigate stress concentration and limit transformation‐induced damage [44]. However, a similar mechanical stability was also observed in the control group, even in the presence of a measurable degraded layer. A comparable behavior has been previously reported for 4Y‐PSZ zirconia, where hydrothermal aging promoted significant tetragonal‐to‐monoclinic transformation without necessarily resulting in a reduction in strength or reliability [30]. In the study by de Assis Marcolino *et al.*, extended hydrothermal aging produced monoclinic fractions close to 40%, yet high mechanical reliability was still observed in single load to failure tests, where strength reduction was limited or material dependent. These findings suggest that phase transformation alone may not be sufficient to compromise mechanical performance unless accompanied by critical flaw growth. Additionally, transformation‐induced compressive stresses may have contributed to maintaining structural integrity, delaying the detrimental effects typically associated with LTD [49, 50].

Fractographic evaluation traced fracture origin to surface defects under tensile stress during fatigue testing, which propagated toward the compressive surface in the control groups. In the glass‐infiltrated group, the fracture likely originated from defects at the interface between the zirconia surface and the glass layer, from which cracks propagated toward the compressive surface. This behavior is similar to that reported for glass infiltration of 3Y‐TZP in the literature [36, 40, 42, 51] and further explains the observed increase in characteristic strength and survival probability in the glass‐infiltrated groups. The absence of SEM‐based fractographic analysis represents a limitation of the present study, which could have provided additional insight into crack initiation and propagation mechanisms within the graded layer. Nevertheless, fracture origin and propagation patterns were consistently identified by polarized light stereomicroscopy and agreed with previously reported behavior for glass‐infiltrated zirconia systems [51, 52, 53]. Despite the valuable insights provided by this study, other limitations must be acknowledged. First, the mechanical evaluation was performed using simplified specimen geometries under cyclic loading, which do not fully reproduce the complex stress distributions, cyclic loading, and tribological interactions present in clinical restorations. Additionally, hydrothermal aging protocols, although widely used to accelerate degradation phenomena, cannot perfectly replicate the combined effects of moisture, mechanical fatigue, thermal fluctuations, and oral chemistry observed in vivo [13]. Therefore, further studies are warranted to investigate the long‐term fatigue behavior of glass‐graded 4Y‐PSZ zirconia, particularly under cyclic loading, anatomical geometries, and thermomechanical aging conditions, as well as to determine the stability of the graded structure over extended simulated clinical service periods. Moreover, the graded structure obtained in the present study was only qualitatively characterized; further evaluations, including nanoindentation mapping, EDS line scanning, or Raman spectroscopy, are recommended to validate the graded nature of the infiltrated structure.

Overall, the findings of the present study indicate that modifying the surface and near‐surface structure of 4Y‐PSZ zirconia through glass infiltration provides a synergistic effect on mechanical reliability by simultaneously influencing flaw population, stress distribution, and on resistance to hydrothermal degradation. The graded structure appears to reduce the severity of critical surface defects and to promote a more favorable stress profile at the tensile surface, which is consistent with the observed increase in characteristic strength and survival probability under cyclic loading. From a clinical standpoint, this approach may contribute to expanding the safe application of translucent zirconias in more demanding restorative scenarios, supporting the ongoing development of monolithic ceramic systems that combine esthetics, durability, and long‐term reliability. In brief, graded glass infiltration improved the fatigue behavior of 4Y‐PSZ zirconia, increasing characteristic strength and survival probability under accelerated lifetime testing. These findings suggest that glass‐graded 4Y‐PSZ may expand the mechanical applicability of translucent zirconia for high‐load prosthetic indications.

## AUTHOR CONTRIBUTIONS

**Conceptualization**: Ernesto Byron Benalcazar‐Jalkh, Estevam Augusto Bonfante, Tiago Moreira Bastos Campos, Larissa Marcia Martins Alves, Paulo Guilherme Coelho, and Lukasz Witek. **Methodology**: Felipe Machado Souza, Larissa Marcia Martins Alves, Tiago Moreira Bastos Campos, Mariana Miranda de Toledo Piza, Satoshi Yamaguchi, Petra Christine Gierthmuehlen, Lukasz Witek, Paulo Guilherme Coelho, Estevam Augusto Bonfante, and Ernesto Byron Benalcazar‐Jalkh. **Software**: Larissa Marcia Martins Alves, Tiago Moreira Bastos Campos, Giovana Assis Marcolino, Mariana Miranda de Toledo Piza, Rainã Sousa Dias, and Ernesto Byron Benalcazar‐Jalkh. **Data curation**: Giovana Assis Marcolino, Mariana Miranda de Toledo Piza, Rainã Sousa Dias, Satoshi Yamaguchi, and Lukasz Witek. **Investigation**: Felipe Machado Souza, Larissa Marcia Martins Alves, Edisa O. Sousa, Tiago Moreira Bastos Campos, Giovana Assis Marcolino, Mariana Miranda de Toledo Piza, Rainã Sousa Dias, and Ernesto Byron Benalcazar‐Jalkh. **Validation**: Felipe Machado Souza, Edisa O. Sousa, Tiago Moreira Bastos Campos, Giovana Assis Marcolino, Rainã Sousa Dias, Satoshi Yamaguchi, Petra Christine Gierthmuehlen, Lukasz Witek, Paulo Guilherme Coelho, and Estevam Augusto Bonfante. **Formal analysis**: Edisa O. Sousa, Giovana Assis Marcolino, Mariana Miranda de Toledo Piza, and Ernesto Byron Benalcazar‐Jalkh. **Supervision**: Larissa Marcia Martins Alves, Tiago Moreira Bastos Campos, Estevam Augusto Bonfante, and Ernesto Byron Benalcazar‐Jalkh. **Funding acquisition**: Estevam Augusto Bonfante and Ernesto Byron Benalcazar‐Jalkh. **Visualization**: Felipe Machado Souza, Edisa O. Sousa, Rainã Sousa Dias, Satoshi Yamaguchi, Petra Christine Gierthmuehlen, and Lukasz Witek. **Project administration**: Estevam Augusto Bonfante and Ernesto Byron Benalcazar‐Jalkh. **Resources**: Estevam Augusto Bonfante and Ernesto Byron Benalcazar‐Jalkh. **Writing—original draft**: Edisa O. Sousa and Giovana Assis Marcolino. **Writing—review and editing**: Larissa Marcia Martins Alves, Tiago Moreira Bastos Campos, Satoshi Yamaguchi, Petra Christine Gierthmuehlen, Lukasz Witek, Paulo Guilherme Coelho, Estevam Augusto Bonfante, and Ernesto Byron Benalcazar‐Jalkh.

## CONFLICT OF INTEREST STATEMENT

The authors declare no conflicts of interest.
